# Exploring the coding rare variants in Alzheimer’s disease

**DOI:** 10.1002/alz.089730

**Published:** 2025-01-03

**Authors:** Po‐Liang Cheng, Wan‐Ping Lee

**Affiliations:** ^1^ Penn Neurodegeneration Genomics Center, Perelman School of Medicine, University of Pennsylvania, Philadelphia, PA USA; ^2^ Department of Pathology and Laboratory Medicine, Perelman School of Medicine, University of Pennsylvania, Philadelphia, PA USA

## Abstract

**Background:**

People leverage single‐variant association test to systematically evaluate common genetic variants (minor allele frequency 0.5% < [MAF) < 5%) for complex disease, such as Alzheimer’s disease (AD). Rare variants (MAF < 1%) could explain additional disease risk and are known to play an important role in the diseases. Unlike common variants, performing traditional single‐variant analysis on rare variants is often underpowered for general study sample sizes. A satisfied power for using single‐variants analysis on rare variants requires extremely large sample sizes that are often economically unaffordable. The alternative method to study the association between AD and rare variants was using aggregative testing that evaluate association for multiple variants in a biologically relevant region, such as a gene. However, the aggregative testing could only determine a set of variants instead of precisely determined the specific causal variant. Therefore, we try to use a new approach to identify coding AD causal variants.

**Method:**

We first explored the case‐only coding rare variants in two independent datasets (ADSP with 16905 whole‐genome‐sequencing samples and ADGC with 38271 genotype samples that imputed by TOPMed). Then, we filtered variants that have at least two carriers in both datasets and CADD > 20. We further utilized STRING to build the protein‐protein interaction network for finding out the relation strength to known AD genes for those variants located in non‐conventional AD genes. Genes of the candidate rare variants were used to construct the protein‐protein interaction network with known AD genes. Non‐conventional AD genes that linked at least to five known AD genes, genes expressed in brain region, or neuron cells were selected and through literature searching to determine as potential novel AD genes.

**Result:**

Through this approach, we identified seven coding rare variants in two known AD genes, SORL1 and CASP7, and in five novel AD genes, W LRP2, SPAG9, LEF1, PI4KA and USH2A. These potentially causal variants of AD could be further validated by biological functional assay in the future.

**Conclusion:**

The new approach could help us to explore rare variants and discovered potential novel genes that might contribute large impact in causing AD.

